# Revitalizing Hair Growth: A New Regimen Utilizing Growth Factor Concentrate for Hair Loss Treatment

**DOI:** 10.7759/cureus.63354

**Published:** 2024-06-28

**Authors:** Aksha Bhargava, Vikas K Singh, Ruchika Tiwari, Akshita Arya, Krupa Chokshi

**Affiliations:** 1 Oral and Maxillofacial Surgery, Mahatma Gandhi Dental College and Hospital, Jaipur, IND

**Keywords:** platelet-rich plasma, hair regrowth, growth factor concentrates, alopecia, hair loss

## Abstract

Androgenic alopecia is a common and major cause of progressive hair loss. Hair loss can be prevented by administrating concentrated growth factor concentrate (GFC) into the scalp where the person's own growth factors are effectively delivered deep within the layers of the scalp, promoting optimal results. This prospective study includes five patients with androgenic alopecia. Growth factor concentrates were injected subcutaneously into the scalp, and assessment was done before the procedure two and eight weeks after the first sitting. The treatment outcome was evaluated with macroscopic and trichoscopic microphotographs. The trichoscopic microphotographs provided further evidence of the notable improvement in hair density. Additionally, the hair pull test yielded negative results during the eight-week follow-up, confirming the positive outcomes of the GFC treatment. Patients were highly satisfied with the results: faster rate of hair growth, better quality of hair, and minimal side effects.

## Introduction

Hair loss is a common problem that requires actions to stop or reduce it. Androgenic alopecia (AGA) is described as progressive hair loss, having a specific distribution pattern. It is a primary cause of hair loss [1]. This condition affects males and females, affecting their self-esteem and confidence and impairing their quality of life [2]. AGA is ascribable to hormonal and genetic factors characterized by a distributed pattern of hair loss. It is a condition that presents with elevated levels of 5-alpha reductase around the hair follicle. This enzyme converts testosterone to dihydrotestosterone, which turns stable hair follicles into unstable hair follicles, weakening them and ultimately leading to hair fall [3]. Fortunately, hair loss can be reversed using the individual’s own platelets without the need for any specialized surgery. Hair replenishment is attained using the innovative approach of growth factor concentrate (GFC) therapy. GFC, meticulously formulated and concentrated from the individual's own blood, produces remarkable results in addressing hair loss and facial rejuvenation. Platelets, a type of blood cell, naturally contain numerous growth factors [1]. Wockhardt Ltd. (Mumbai, India) has developed a method for extracting and concentrating these growth factors from the patient's blood, resulting in a potent GFC formulation through purpose-built kits.

## Case presentation

**Figure 1:**
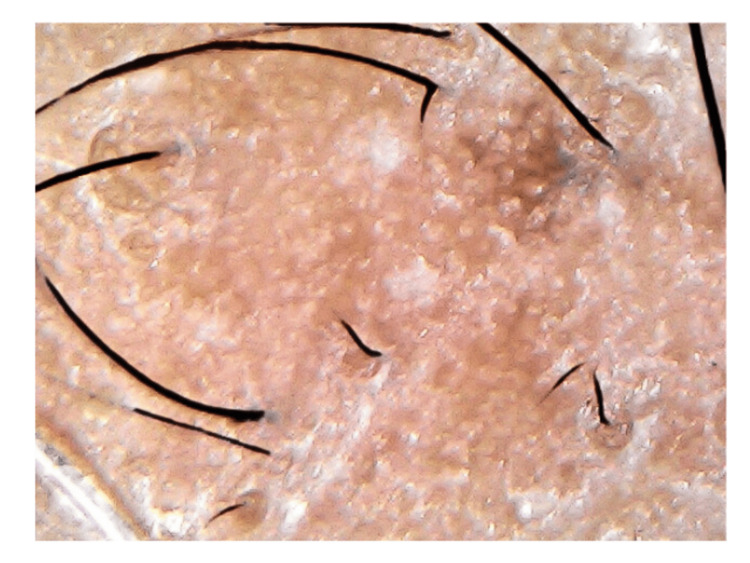
Dermoscopic preoperative picture showing hair density

Five patients were included in our study with various grades of AGA. The patients' age ranges from 20 to 30 years. All patients were male. The grading of alopecia was done by a single observer and was based on the Norwood‑Hamilton scale for alopecia. This grading system involves staging from I to VII and A represents alopecia from the anterior region They had complained of hair loss, decreased hair density, and most commonly hair fall, and they gave no history of taking or applying any medications for hair loss. None of the patients reported a history of systemic diseases or keloidal tendencies. Preoperative pictures were taken with a dermoscope (Figure 1).

Preparation of GFC

**Figure 2:**
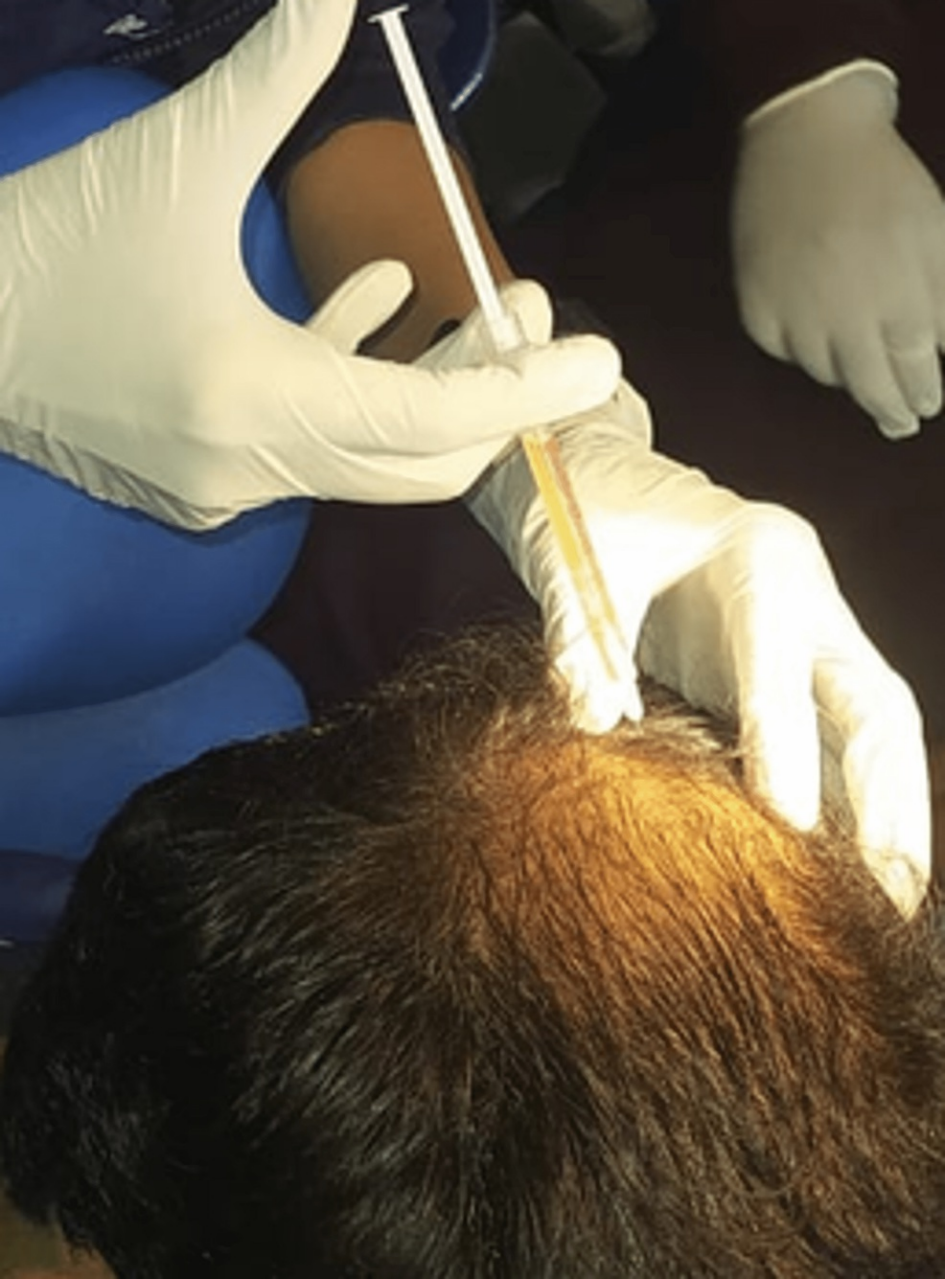
Injecting growth factor concentrate (GFC) using an insulin syringe

To prepare for the treatment, 20 to 25 ml of each individual’s peripheral blood was collected and equally distributed in four GFC tubes (i.e., vacuettes). The peripheral blood was mixed by inverting the tubes 6 to 10 times and then allowing them to stand for approximately 30 minutes. The peripheral blood was then injected using a syringe (Figure 2). The GFC treatment utilizes Wockhardt’s proprietary platelet-activating solution contained within the tube, effectively activating the platelets and releasing plasma-derived growth factor, vascular endothelial growth factor, epidermal growth factor, insulin-like growth factor, and other growth factors. Tubes were centrifuged at 3400 rpm for 10 minutes, assisting with counterbalancing. In addition, it is beneficial to separate pure growth factors from red blood cells, white blood cells, and other blood cells present in them [2,4,5].

Treatment and follow-up

During the spins, the scalp is anesthetized using a mixture of xylocaine and vasoconstrictor-like adrenaline in the concentration of 1:200,000, and supraorbital and supratrochlear nerve blocks are given, thus preparing the scalp for injections. The collected growth factor concentrates were then injected into the scalp using an insulin syringe in the galea aponeurotica according to of hair loss area. Four sessions were done over eight weeks, keeping an interval of two weeks, and receiving five to seven injections per session.

Outcome and evaluation

**Figure 3:**
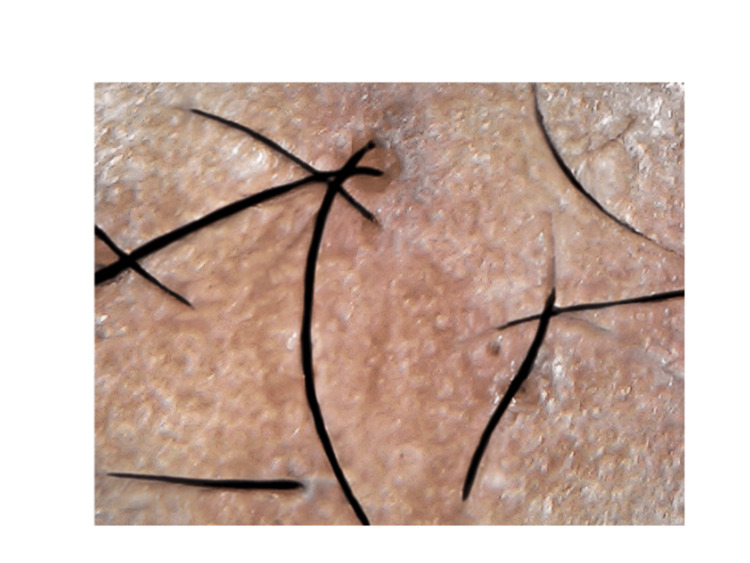
Two-week follow-up

**Figure 4:**
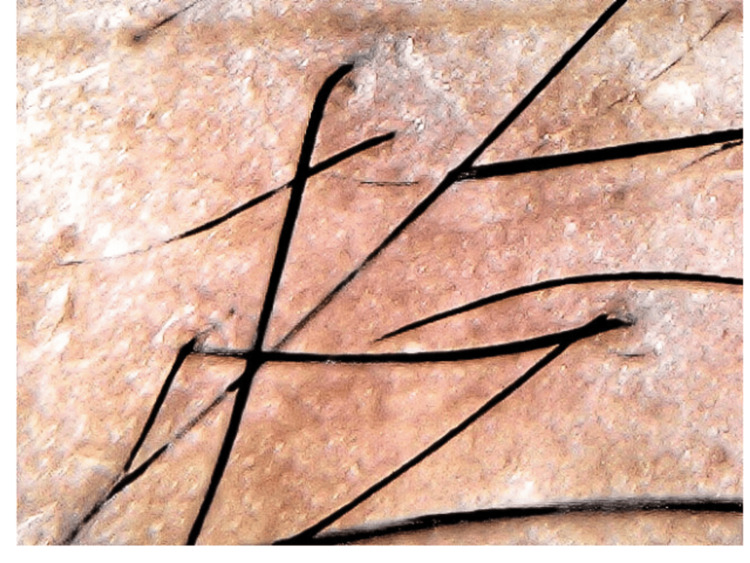
Eight-week follow-up

Hair status was assessed with preoperative and postoperative dermascope photographs at the two-week (Figure 3) and eight-week (Figure 4) follow-ups, and results were evaluated using a hair pull test and the change in hair density at the treated site.

Results

**Figure 5:**
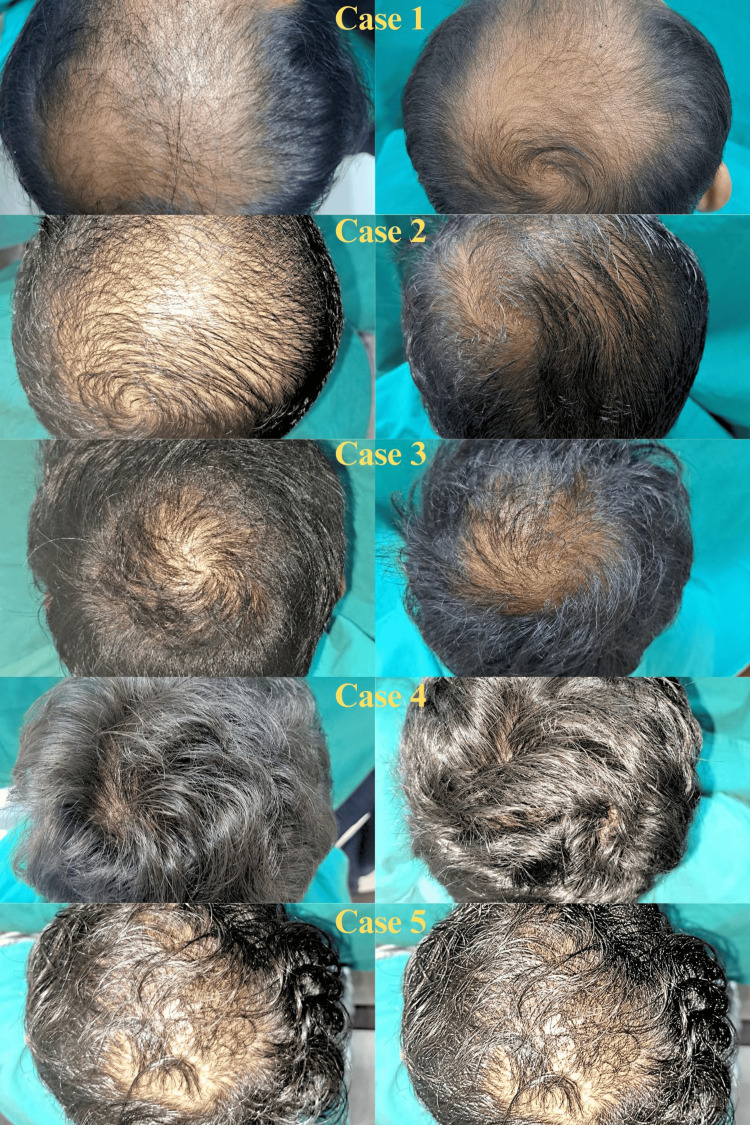
Pre (left) and post (right) GFC treatment photograph GFC: growth factor concentrate

All five patients were evaluated every two weeks, and the pictures taken before and eight weeks after treatment were compared. Table 1 gives a brief of all patients. The mean age of the patients was 23.6 years (22 to 29 years). To grade alopecia, the Norwood Hamilton classification was used. Two patients suffered from Grade II, one from Grade III, one from Grade IV, and one from Grade V alopecia. Two patients with Grade II alopecia had the main complaint of hair fall. All the patients underwent GFC therapy and were evaluated. Macroscopic photographs revealed noticeable enhancement in the hair quality and density, transforming thin, lanugo-like hair into thick, healthy hair strands. GFC treatment demonstrated remarkable refinement in the appearance of hair, improved hair density, and reduced hair loss significantly. There was no infection, redness, or itching at the site of injection. The trichoscopic microphotographs provided further evidence of the notable improvement in hair density. Additionally, a hair pull test yielded negative results during the eight-week follow-up, confirming the positive outcomes of the GFC treatment. Figure 5 shows all the patients' pre and post-GFC therapy results.

**Table 1:** Patient characteristics

Case	Age (Years)	Alopecia Grade
1	29 years	Grade V
2	23 years	Grade IV
3	22 years	Grade III
4	22 years	Grade II
5	22 years	Grade II

## Discussion

Various treatment options have been proposed to address androgenic alopecia, including the use of growth concentrates, platelet-rich plasma, and other growth factors. In recent years, there has been growing interest in these treatments due to their potential to stimulate hair growth and improve hair density. This interest has been fueled by studies such as that of Dhurat et al. [4], which demonstrated the efficacy of micro-needling in conjunction with platelet-rich plasma in treating AGA. Other studies, as done by Kim et al. [5] and Khatu et al. [6], have scrutinized the use of platelet-rich plasma and GFC, respectively, as standalone treatments for AGA. Moreover, Fertig and Gamret [7] have reviewed the current evidence for the use of platelet-rich plasma and growth factors in treating AGA, providing valuable insights into their efficacy and safety. These studies highlight the potential benefits of GFC and other growth factor treatments in addressing hair loss and provide a basis for further research in this area. Hair loss, especially AGA, is a prevalent issue that affects both men and women. Numerous factors contribute to this condition, including heightened stress levels, hormonal imbalances, genetic conditions, and deficiencies in various essential nutrients [8]. The current conventional treatment approach primarily involves the use of oral or topical medications. However, these treatments necessitate long-term medication usage, and their effectiveness is often hindered by challenges related to patient adherence and the frequent occurrence of side effects. Surgical interventions, such as hair transplantation, are typically reserved for patients who have areas with no hair follicles. Given the varying levels of efficacy and safety associated with existing treatment modalities, there is an ongoing demand for additional interventions that effectively promote hair growth. This groundbreaking GFC treatment involves the precise injection of a person’s own growth factors (e.g., epidermal growth factor vascular, endothelial growth factor, platelet-derived growth factor, and insulin-like growth factor 1) directly into the scalp. This targeted delivery at the hair root promotes the stimulation of hair regrowth by providing these growth factors in a concentrated form. Blood platelets are enriched with various growth factors. Scientists extract them from the blood at a high concentration using a specially designed GFC kit. Specialists then administer the collected GFC with precise tools. The overall process involves no platelet loss. It is nonpyrogenous and secure, and it regenerates damaged tissues naturally. This advanced hair loss treatment helps a person achieve optimal results in only three to four sittings. Therefore, it is the best procedure to restore lost hair. Various growth factors are stored in blood platelets, which help regenerate and repair the tissues and stem cell proliferation, migration, and differentiation. Thus, each GFC plays a unique role in fighting hair loss [9].

Platelet-derived growth factor (PDGF): This helps enhance hair growth by stimulating vascularization and angiogenesis. It also helps in the mitogenesis of endothelial cells and mesenchymal stem cells and the chemotaxis and proliferation of fibroblasts.

Vascular endothelial growth factor (VEGF): This is expressed by dermal papilla (DP) cells during the anagen phase of hair growth and plays a vital role in regulating perifollicular angiogenesis. Additionally, it enhances the size of perifollicular vessels, thereby facilitating a seamless progression of the anagen growth phase.

Epidermal growth factor (EGF): This serves as a catalyst for hair cell proliferation and regeneration, stimulating the growth and division of both mesenchymal and epithelial cells. Moreover, it acts in conjunction with other factors to promote angiogenesis, fostering the development of new blood vessels.

Insulin-like growth factor-1 (IGF-1): This helps maintain the growth of hair follicles, increasing overall hair growth and working as an angiogenesis stimulator.

These growth factors act on the hair follicle stem cells, thus promoting neovascularization and the growth of new follicles. PDGF forms the hair canal, VEGF promotes angiogenesis, EGF promotes the proliferation of the hair shaft and aids hair growth, and IGF-1 promotes follicular growth [10].

## Conclusions

The use of GFC and platelet-rich plasma has shown promising results and may be considered a treatment option for AGA. Studies have demonstrated the potential efficacy of these treatments in stimulating hair growth and improving hair density in AGA patients. The mechanisms underlying these effects are not fully understood but may involve the promotion of angiogenesis and the release of growth factors that promote hair growth. GFC helps in the reduction of hair loss, improves hair thickness, and enhances hair volume. Although further research is needed to establish the efficacy and safety of these treatments, the current evidence suggests they may be a viable option for people looking to address hair loss. It is crucial for patients to consult with a qualified healthcare professional before pursuing these treatments to ensure they are appropriate and safe for their situation. Overall, the use of GFC in treating hair loss is a potential area of research that may provide patients with a safe and effective option for addressing this common concern.
